# Green Synthesis of Zinc Oxide Nanoparticles (ZnO-NPs) Using *Arthrospira platensis* (Class: Cyanophyceae) and Evaluation of their Biomedical Activities

**DOI:** 10.3390/nano11010095

**Published:** 2021-01-04

**Authors:** Ehab F. El-Belely, Mohamed M. S. Farag, Hanan A. Said, Abeer S. Amin, Ehab Azab, Adil A. Gobouri, Amr Fouda

**Affiliations:** 1 Botany and Microbiology Department, Faculty of Science, Al-Azhar University, Cairo 11884, Egypt; elbelely@azhar.edu.eg (E.F.E.-B.); mohamed.farag@azhar.edu.eg (M.M.S.F.); 2 Botany Department, Faculty of Science, Fayoum University, Fayoum 63511, Egypt; hah01@fayoum.edu.eg; 3 Botany Department, Faculty of Science, Suez Canal University Ismailia, Ismailia 41522, Egypt; abeeramin2003@yahoo.com; 4 Department of Biotechnology, College of Science, Taif University, P.O. Box 11099, Taif 21944, Saudi Arabia; e.azab@tu.edu.sa; 5 Botany and Microbiology Department, Faculty of Science, Zagazig University, Zagazig 44519, Egypt; 6 Department of Chemistry, College of Science, Taif University, P.O. Box 11099, Taif 21944, Saudi Arabia; a.gobouri@tu.edu.sa

**Keywords:** cyanobacteria, *Arthrospira platensis*, ZnO-NPs, antimicrobial, in vitro cytotoxicity

## Abstract

In this study, zinc oxide nanoparticles (ZnO-NPs) were successfully fabricated through the harnessing of metabolites present in the cell filtrate of a newly isolated and identified microalga *Arthrospira platensis* (Class: Cyanophyceae). The formed ZnO-NPs were characterized by UV–Vis spectroscopy, Fourier transform infrared (FT-IR)**,** transmission electron microscopy (TEM), energy-dispersive spectroscopy (EDX), X-ray diffraction (XRD), and X-ray photoelectron spectroscopy (XPS). Data showed the efficacy of cyanobacterial metabolites in fabricating spherical, crystallographic ZnO-NPs with a size ≈30.0 to 55.0 nm at a wavelength of 370 nm. Moreover, FT-IR analysis showed varied absorption peaks related to nanoparticle formation. XPS analysis confirms the presence of Zn(II)O at different varied bending energies. Data analyses exhibit that the activities of biosynthesized ZnO-NPs were dose-dependent. Their application as an antimicrobial agent was examined and formed clear zones, 24.1 ± 0.3, 21.1 ± 0.06, 19.1 ± 0.3, 19.9 ± 0.1, and 21.6 ± 0.6 mm, at 200 ppm against *Bacillus subtilis*, *Staphylococcus aureus*, *Pseudomonas aeruginosa*, *Escherichia coli*, and *Candida albicans*, respectively, and these activities were reduced as the NPs concentration decreased. The minimum inhibitory concentration (MIC) values were determined as 50 ppm for *S. aureus,* 25 ppm for *P. aeruginosa*, and 12.5 ppm for *B. subtilis*, *E. coli*, and *C. albicans.* More interestingly, ZnO-NPs exhibit high in vitro cytotoxic efficacy against cancerous (Caco-2) (IC_50_ = 9.95 ppm) as compared with normal (WI38) cell line (IC_50_ = 53.34 ppm).

## 1. Introduction

Nanotechnology is a multidisciplinary science concerning producing novel materials at the nano-scale size (1–100 nm), which can be integrated into various applications [1]. At the nanoscale, the materials acquired new features such as large surface area, thermal conductivity, size, charge, shape, crystal structure, surface morphology, and zeta potential, which enables them to integrate into biomedical and biotechnological sectors [2,3,4]. Nanoparticles (NPs) can be fabricated by different methods including chemical, physical, and biological methods. The former chemical and physical methods utilized hazardous material, needed harsh conditions such as temperature, energy, and pressure, and they can produce hazardous by-products [2,5]. Therefore, the interest increased in biological methods or green nanotechnology.

Green nanotechnology means a clean method utilized for the synthesis of nanomaterials by eliminating or decreasing hazardous materials used during the fabrication process [6]. The green synthesis of NPs can be accomplished using various biological entities such as bacteria, actinomycetes, fungi, cyanobacteria, macro-algae, and plants [7]. Green synthesis is preferred over chemical and physical methods because of its eco-friendly, cost-effectiveness, easy handling, upscaling, and biocompatibility [6]. Recently, several NPs are synthesized by green methods such as Ag, Au, Cu, CuO, ZnO, Se, and others that are integrated into different biological activities [8,9,10]. Biogenic or green synthesized nanoparticles offer a promising alternative antimicrobial and anticancer agent for more safe, specific, and economic drugs or drug vehicle in drug delivery. 

The biogenic synthesis of zinc oxide nanoparticles (ZnO-NPs) by biological entities are attributed to the presence of different metabolites including proteins, enzymes, and other biomolecules that act as reducing, capping, and stabilizing agents. The different shape, size, dispersity of ZnO-NPs, and stability are related to the secreted metabolites [11]. The extracellular mechanisms of ZnO-NPs can be accomplished by the reductase enzyme secreted by microbes in growth media. NADH (Nicotinamide Adenine Dinucleotide plus hydrogen ion)-dependent reductase enzymes serve as electron carriers to reduce Zn^2+^ to Zn^o^, which subsequently forms ZnO-NPs [12]. The reductase enzymes obtained electrons from NADH that oxidized after that to NAD^+^; at the same time, metal ions reduced to nanoscales.

Zinc oxide nanoparticles (ZnO-NPs) are considered the most significant between the metal oxides NPs due to their unique chemical and physical properties, which hence increase their applicability aspects [13]. ZnO-NPs can be integrated into the rubber industry because they furnish wearproof composites and increase the intensity and toughness of the rubber [14]. In addition, ZnO-NPs interweave with sunscreen and cosmetic care products due to their highly UV-adsorption properties [15]. Moreover, due to the unique properties that arise at the nanoscale structure such as high electron mobility, wide bandgap, and high visible transparency, ZnO-NPs are considered a good semiconductor. In the textile industry, ZnO-NPs are added to finished fabrics to increase their resistance to ultraviolet rays, antibacterial, and deodorant activities [16]. As a part of the applications, ZnO-NPs can be used for antifungal, concrete production, solar cell, electronics, photocatalysis, and electrotechnology industries [17,18]. Recently, ZnO-NPs have been validated as additives to dietary products to improve the growth performance, enhance the antioxidant property and immune response, and increase the quality of eggs and improve the production of layer chickens [19].

Zinc is an essential trace present in all body tissues, in addition to being a constant part of most enzyme systems, so it participates in the body’s metabolism process and is assimilated during nucleic acid and protein synthesis, nervous cell synthesis, and hematopoiesis [17]. At the nanoscale, ZnO-NPs can be adsorbed easily especially at a small size. Interestingly, ZnO-NPs can be used in the food industry as additives, especially since they are recommended as a safe substance by the FDA (Food and Drug Administration) [20], meaning that they have safe applicability for human and animals. 

Cyanophyceae are a group of prokaryotic structured cells that possess carbon dioxide-dependent photosynthesis [21]. They are considered a good source for the extracellular and intracellular synthesis of NPs due to their potential to produce a huge number of metabolites [21]. Various NPs are fabricated through harnessing metabolites of cyanobacteria such as Ag, Au, Pt, and Pd-NPs [22,23]. The most common cyanobacterial species used for the fabrication of NPs are *Spirulina* spp. and *Nostoc* spp. due to the high contents of bioactive substances [24]. Amongst cyanobacterial species, *Arthrospira platensis*, previously called *Spirulina*, is a planktonic multicellular filamentous cyanobacterium that commonly grows in subtropical, alkaline fresh, and marine aquatic habitats. The edible biomass of *A. platensis* is a rich source of nutraceutical and pharmaceutical biomolecules such as proteins, vitamins, pigments, and polysaccharides as well as minerals [25], and it frequently can be used for the ecofriendly biogenic synthesis of metallic and metal oxide nanoparticles by either extracellular or intracellular mechanisms. Moreover, the rapid growth rates, avoidance of contaminations as no complicated nutrients were added to the media, and low cost of biomass production make *A. platensis* a promising source for the biosynthesis of nanoparticles. [26].

This study aims to investigate the fabrication of ZnO-NPs using a newly isolated and characterized microalga, *A. platensis.* Characterizing the green biosynthesized NPs using UV–Vis spectroscopy, Fourier transform infrared (FT-IR), XRD, TEM, energy-dispersive spectroscopy (EDX), and XPS analyses is another major goal. In addition, evaluation of the biological activities of green synthesized ZnO-NPs including antimicrobial activity against pathogenic Gram-positive and Gram-negative bacteria as well as unicellular fungi and investigating their in vitro cytotoxic effect against normal and cancerous cells were investigated for possible application in the biomedical field.

## 2. Materials and Methods 

### 2.1. Isolation, Purification, and Identification of Cyanobacterial Strain

The cyanobacterial strain was isolated from a water sample collected from EL-Kanater El-Khairia, Dakahlia governorate, Egypt, and transferred directly to Algal Lab., Botany and Microbiology Department, Faculty of Science, Al-Azhar University, Cairo, Egypt. The isolation procedure was achieved by cultivated in Zarrouk’s media and incubated at 30 ± 1 °C with a photoperiod 8/16 h of dark/light cycle using cool-white, fluorescent lamps at 3000 Lux of light intensity. The appeared colony was checked for purity, and the purified isolate was preserved for further study. The morphological characteristics were examined using a light microscope, which was used to check the purity, shape, color, and structure of cyanobacterial isolate. The microscopic taxonomy was achieved according to Edlund [27].

On the other hand, the molecular identification based on 16S rRNA of cyanobacterial strain was achieved as the following: genomic DNA of cyanobacteria sp. was extracted according to the method recommended by Miller et al. [28]. Briefly, purified colonies were resuspended in 50 μL of sterile deionized H_2_O. The cyanobacterial cell suspension is heated in a water bath at 97 °C for 10 min; after that, it was centrifuged at 15,000 rpm for 10 min, and the cyanobacterial cell lysate containing the DNA was obtained. Then, 16S rRNA was amplified in a polymerase chain reaction (PCR) using the genomic DNA as a template and universal primers, 27f (5-AGAGTTTGATCCTGGCTCAG-3) and 1492r (5-GGTTACCTTGTTACGACTT-3) [29]. The PCR mixture (50 μL) contained the following: 1× PCR buffer, 0.5 mM MgCl_2_, 2.5 U Taq DNA polymerase (QIAGEN), 0.25 mM dNTP, 0.5 μM of each primer, and 1 μL of extracted cyanobacterial genomic DNA. 

The PCR was analyzed in a DNA Engine Thermal Cycler by Sigma Scientific Services Company (Cairo, Egypt) with a hot starting at 94 °C for 3 min, followed by 30 cycles of 94 °C for 30 s, 55 °C for 30 s, and 72 °C for 1 min, followed by a final extension at 72 °C for 10 min. Sequencing was conducted using ABI 3730x1 DNA sequencer at GATC Company (Konstanz, Germany). The obtained sequences were compared against the Gene Bank database using the NCBI BLAST (Basic Local Alignment Search) program. Then, sequences were compared with 16S rRNA in the Gene Bank database using BLASTN, and phylogenetic trees were conducted by bootstrap analysis.

### 2.2. Cyanobacterial Mediated Green Synthesis of ZnO-NPs

#### 2.2.1. Biomass Preparation

The cyanobacterium strain, *A. platensis* (EF), was grown in Zarrouk’s medium supplied with filtered air under the previously mentioned condition to prepare cyanobacterial biomass.

#### 2.2.2. Cell Filtrate-Mediated Biosynthesis of ZnO-NPs

Microalga biomass was utilized in the logarithmic phase. The cultivated strain was subjected to a centrifugation process to separate the microalga biomass and then washed thrice with double-distilled deionized water to remove any medial components. After that, the washed biomass (15 g) was mixed with 100 mL of distilled water for 48 h and underwent a centrifugation process to collect the biomass filtrate (supernatant without any biomass). ZnO nanoparticles were synthesized as follows: 0.44 g of Zn(CH_3_COO)_2_· 2H_2_O was dissolved in two mL of distilled H_2_O; after that, 98 mL of biomass filtrated was added to get a final concentration of 2 mM. The mixture was incubated for 24 h at 30 °C ± 2 °C and 150 rpm shaking condition. The resultant white precipitate was collected and oven-dried at 200 °C for 24 h [30] to obtain ZnO-NPs as a powder, which was used after that for further study.

### 2.3. Characterization of Green Synthesized ZnO-NPs 

#### 2.3.1. Ultraviolet-Visible (UV–Vis) Spectra

The formation of ZnO-NPs was investigated by mixture solutions color changes. The green synthesized ZnO-NPs in a colloid solution was also monitored by UV–Vis spectra, as it showed an intense absorption peak due to surface plasmon excitation. Color changes in the mixture of cyanobacterial extract/Zn(CH_3_CO_2_)_2_ solutions were measured by Spectrophotometry (JENWAY 6305 Spectrophotometer, 230 V/50 Hz, Staffordshire, UK), at wavelengths of 200–800 nm. The cyanobacterial extract without Zn(CH_3_CO_2_)_2_ was used as the blank.

#### 2.3.2. Fourier Transform Infrared Spectroscopy (FT-IR)

The functional groups present in green synthesized ZnO-NPs were analyzed by FT-IR analysis (JASCO FT-IR 4100 spectrometer, Hachioji, Tokyo, Japan). About 0.2 g of ZnO-NPs powder was mixed with potassium bromide (KBr) and loaded onto a disc at high pressure. The FT-IR spectra were scanned at a resolution of 4.0 cm^−1^ at a wavelength of 400–4000 cm^−1^.

#### 2.3.3. Transmission Electron Microscopy (TEM) and Energy Dispersive Spectroscopy (EDX)

The morphological analysis including the shape and size of cyanobacterial mediated biosynthesized of ZnO-NPs were examined by TEM (JEM-1230, JEOL, Tokyo, Japan). The sample was prepared by adding a drop of colloidal ZnO-NPs solution on a copper grid covered by an amorphous carbon film and desiccating the solvent under vacuum overnight before loading onto a specimen holder. AMT software was calibrated for NPs size measurements by a digital TEM camera. The average size of the fabricated ZnO-NPs was calculated from measuring over 100 nanoparticles in at least 10 random locations on the TEM grid in enlarged microphotographs. 

The elemental structures of the green synthesized ZnO-NPs were measured by SEM (JEOL JSM-6360LA, Tokyo, Japan), which was connected with an energy-dispersive spectroscope (EDX, Tokyo, Japan) to detect the surface shape and elemental compositions of different the NPs [31].

#### 2.3.4. X-ray Diffraction (XRD) Patterns

The crystalline nature of the ZnO-NPs synthesized by cyanobacterium species was detected by X-ray diffraction (XRD, X’Pert Pro Philips, Dandong, China) at the following operation system: CuKα radiation, the 2*θ* angle was in a range from 0° to 90°, *λ* = 1.540 Å (Eindhoven, Netherlands). The voltage and current were adjusted to 40 kV and 30 mA, respectively. The average nanoparticle size was calculated using the following the Debye–Scherrer Equation (1) [32], shown below:

(1) D = 0.9λ/βCosθ     

where *D* is the average nanoparticle size and 0.9 is the Scherrer’s constant. *λ*, *β*, and *θ* are the X-ray wavelength, Full-Width Half Maximum, and the Bragg’s angle, respectively.

#### 2.3.5. X-ray Photoelectron Spectroscopy (XPS) Analysis

ESCALAB 250XI (Thermo Fischer Scientific Inc., Waltham, MA, USA) equipped with a monochromatic X-ray Al Kα radiation (1486.6 eV) was used for the XPS analysis. For the analysis, the samples were prepared under the pressure of 10^−8^ mbar, and the energy was calibrated with an Ag *3d_5/2_* signal (ΔBE: 0.45 eV) and C *1s* signal (ΔBE: 0.82 eV). The size of the spot was 500 μm and the full and narrow-spectrum pass energies were 50 and 20 eV, respectively [33,34].

### 2.4. Biological Activities of Cyanobacterium-Mediated Green Synthesis of ZnO-NPs

#### 2.4.1. Antimicrobial Activity

The antimicrobial efficacy of green synthesized ZnO-NPs formed through the harnessing of cyanobacterium metabolites was assessed by the agar well diffusion method against prokaryotic and eukaryotic pathogenic species. The prokaryotic species are represented by Gram-positive bacteria (*Staphylococcus aureus* ATCC6538 and *Bacillus subtilis* ATCC6633) and Gram-negative bacteria (*Escherichia coli* ATCC8739 and *Pseudomonas aeruginosa* ATCC9022), while eukaryotic pathogenic microbe represented by unicellular fungi *Candida albicans* ATCC10231. Briefly, each bacterial and unicellular fungal strains (100 μL/1.0 O.D.) were seeded on 100 mL of Mueller Hinton agar media under aseptic condition. After that, 100 μL of stock ZnO-NPs solution (200 ppm) was put into well (0.7 mm) on Mueller–Hinton agar plates. To detect the minimum inhibitory concentration of ZnO-NPs (MIC) for each tested organism, different concentrations (150, 100, 50, 25, 12.5, and 6.25 ppm) were prepared. The inoculated plates were kept in the refrigerator for about 2 h and transferred to the incubator at 35 °C ± 2 °C for 24 h. The results were as the diameter of inhibition zones (mm) around each well [35]. The experiment was done in triplicate. 

#### 2.4.2. In Vitro Cytotoxic Efficacy of ZnO-NPs Synthesized by Cyanobacterium Species

The human WI38 cell line (lung normal cell line) and Caco-2 cancer cell (human colorectal adenocarcinoma cells) were obtained from the American Type Culture Collection (ATCC, Manassas, VA, USA). The efficacy of cyanobacterium-mediated ZnO-NPs synthesis as cytotoxic effects was assessed using an MTT (3-(4,5-dimethylthiazol-2-yl)-2,5-diphenyl tetrazolium bromide) assay against the previous normal WI38 cells and Caco-2. Briefly, the cells were inoculated in 96-well microtiter plates at 1 × 10^5^ cells/well followed by treatment with double-fold dilution (200–6.25 ppm) of the ZnO-NPs and incubated at 37 °C for 48 h. After that, the MTT (5 mg mL^−1^ in phosphate-buffered saline) was added to each inoculated well and incubated for 1–5 h at 37 °C and 5% CO_2_. At the end of the incubation period, the purple formazan crystals were formed, which were further dissolved by adding DMSO (10%). The plates were agitated using a plate shaker for 30 min in dark conditions. Ultimately, the color intensity of samples was measured at 560 nm using a multi-well ELISA plate reader [36]. The cell viability percentage was calculated as follows.

(2) $$\mathrm{Cell}\;\mathrm{viability}\;\left(\%\right)=\;\frac{\mathrm{Absorbance}\;\mathrm{of}\;\mathrm{treated}\;\mathrm{sample}}{\mathrm{Absorbance}\;\mathrm{of}\;\mathrm{control}}\;\times 100$$

### 2.5. Statistical Analysis

One-way analysis of variance (ANOVA) was used to investigate the efficacy of ZnO-NPs as antimicrobial and in vitro cytotoxicity. A posteriori pairwise-multiple comparisons were done using Tukey’s range tests at *α* = 0.05. All results are the means of three independent replicates. Data were statistically analyzed using SPSS v17 (SPSS Inc., Chicago, IL, USA).

## 3. Results and Discussion

### 3.1. Identifications of Cyanophyceae Strain

**Figure 1:**
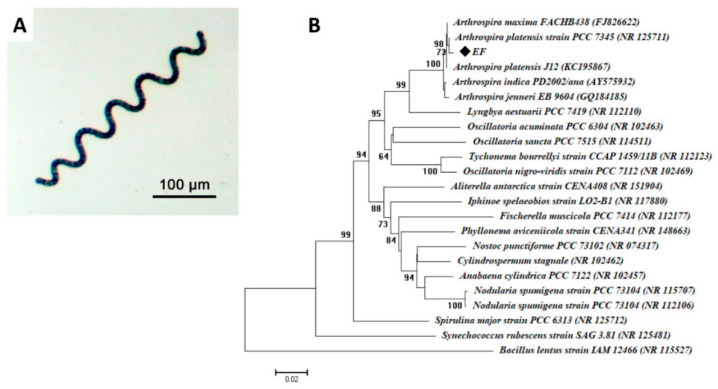
Microscopic and molecular identification of cyanobacteria *Arthrospira platensis* strain (EF). (**A**) denotes a typical single trichome of *A. platensis* (Class: Cyanophyceae); (**B**) denotes a phylogenetic analysis of 16S rRNA sequences of cyanobacterial strain with reference sequences from NCBI. ♦ refers to 16S rRNA sequences of *A. platensis*. The analysis was performed in MEGA6 using the neighbor-joining method.

The light microscope confirms the purity of the isolate, which appeared as a filamentous structure, and the helix, which was open left-handed (Figure 1A). The gene sequence analysis revealed that the cyanobacterial isolate was related to *Arthrospira platensis* (accession number: NR125711) with a similarity percent of 99.55%. The obtained sequence retrieved from this study was a deposit in GeneBank under accession number MW115140. Therefore, the selected cyanobacterial strain was identified as *Arthrospira platensis* strain (EF) (Figure 1B). 

### 3.2. Arthrospira Platensis Mediated Biosynthesis of ZnO-NPs

**Figure 2:**
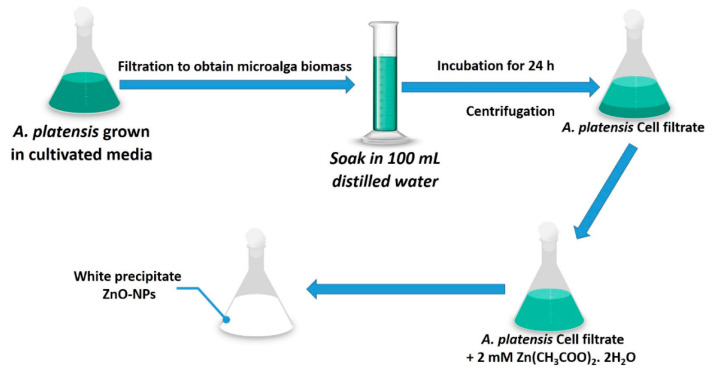
Flowchart of green synthesis zinc oxide nanoparticles (ZnO-NPs) by cyanobacterial *A. platensis*.

The biosynthesis of ZnO-NPs was based on reducing, capping, and stabilizing of precursor (Zn(CH_3_COO)_2_·2H_2_O) by metabolites involved in the cell filtrate of *A. platensis* which contains polysaccharide, proteins, and enzymes (Figure 2). The function of capping agents was preventing the biosynthesis of hydrated ZnO. In this study, the green synthesis of ZnO-NPs was achieved by mixing 2 mM of the precursor with 100 mL of *A. platensis* cell filtrate and incubated at 150 rpm shaking state at room temperature. The as-formed white precipitate was collected and calcinated at 200 °C for 24 h.

(3) $$\mathrm{Zn}{\left({\mathrm{CH}}_{3}\mathrm{COO}\right)}_{2}\cdot 2H_{2}O\;\overset{A.\;platensis\;\mathrm{cell}\;\mathrm{filtrate}}{\to }\;\mathrm{Zn}\left(\mathrm{II}\right)O$$

### 3.3. Characterization of Green Synthesized ZnO-NPs 

#### 3.3.1. UV–Vis Spectroscopic Analysis

**Figure 3:**
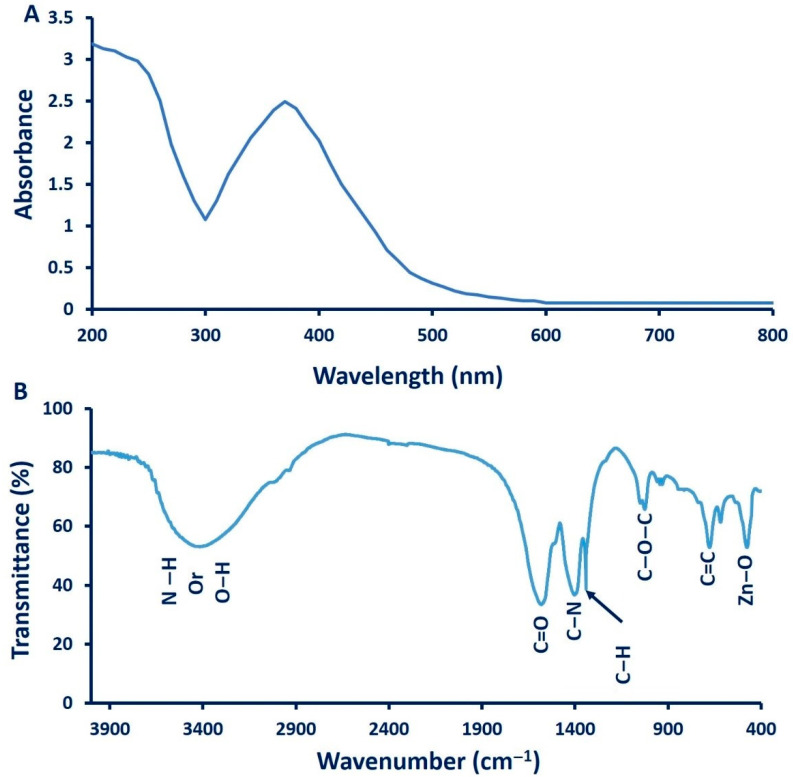
Characterization of green synthesized ZnO-NPs by *A. platensis*. (**A**) UV–Vis spectra, (**B**) Fourier transform infrared (FT-IR) analysis.

The first indicator for ZnO-NPs formation is visual observation followed by measuring this change by UV at various wavelengths (200 to 800 nm) to detect the surface plasmon resonance (SPR). In this study, the color of the cyanobacterial extract was changed to turbid white after adding zinc acetate as a precursor for ZnO-NPs. Figure 3A showed that the UV–Vis spectra of green synthesized ZnO-NPs demonstrated a significant peak of 370 nm, which is mostly distinguished by ZnO-NPs. The obtained results are completely consistent with Singh et al. [37], who reported the efficacy of *Anabaena* strain L3 to fabricate ZnO-NPs and exhibit intense SPR at 370 nm. These findings are incompatible with our previous study of biosynthesized ZnO-NPs using fungal strains *Fusarium keratoplasticum* A1-3 and *Aspergillus niger* G3-1, which exhibit SPR at 390 nm [38]. Vennila and Jesurani [39] reported that the SPR of green synthesized ZnO-NPs was mostly in the wavelength range between 370 and 400 nm.

#### 3.3.2. Fourier Transform Infrared Spectroscopy (FT-IR) Analysis

The functional groups, as well as the chemical structures of *A. platensis*-mediated ZnO-NPs synthesis was determined by FT-IR analysis (Figure 3B). The FT-IR spectra exhibit seven intense peaks at 3415, 1600, 1410, 1341, 1025, 676, and 503 cm^−1^. The broad absorption peak at 3415 cm^−1^ is related to N–H overlapped with a stretching O–H band [33]. The breadth of this broad peak could be attributed to the formation of intra- and intermolecular hydrogen bonds [40]. The low-intensity peak observed at 3000 cm^−1^ is related to the stretching CH_2_ of asymmetric and symmetric carbohydrates and/or lipids [41], whereas the band at 1600 cm^−1^ is corresponding to the stretching C=O vibration of proteins [42] or remaining acetate. The absorption wave of CH_2_ or CH_3_ of proteins is responsible for the vibration bending of the C–H at wavenumber 1341 cm^−1^ [40]. The observed band at 1410 cm^−1^ has corresponded to the C–N stretching bond of amino acid, whereas the band observed at wavelength 1025 cm^−1^ can be attributed to C–O–C ether of polysaccharides [33,43]. The successful formation of Zn–O was confirmed by the absorption band observed at 503 cm^−1^. Consistent with our data, the FT-IR analysis of green synthesized ZnO-NPs showed the Zn–O absorption band has been observed at wavelength 485 cm^−1^ [44], 442 cm^−1^ [45], in range 400 to 500 cm^−1^ [46], or at wavelength 782 cm^−1^ [47], 450 cm^−1^, and 600 cm^−1^ [48]. The data of FT-IR analysis exhibit the role of organic substances in *A. platensis* extract in the reduction, capping, and stabilizations of biosynthesized ZnO-NPs. Azizi et al. [49] suggested that the formation of ZnO-NPs was accomplished as a result of the interaction between oxygen in functional groups involved in cell extract of *Sargassum muticum* and zinc molecules in salt precursors.

#### 3.3.3. Transmission Electron Microscopy (TEM) and Energy-Dispersive Spectroscopy (EDX) Analysis

**Figure 4:**
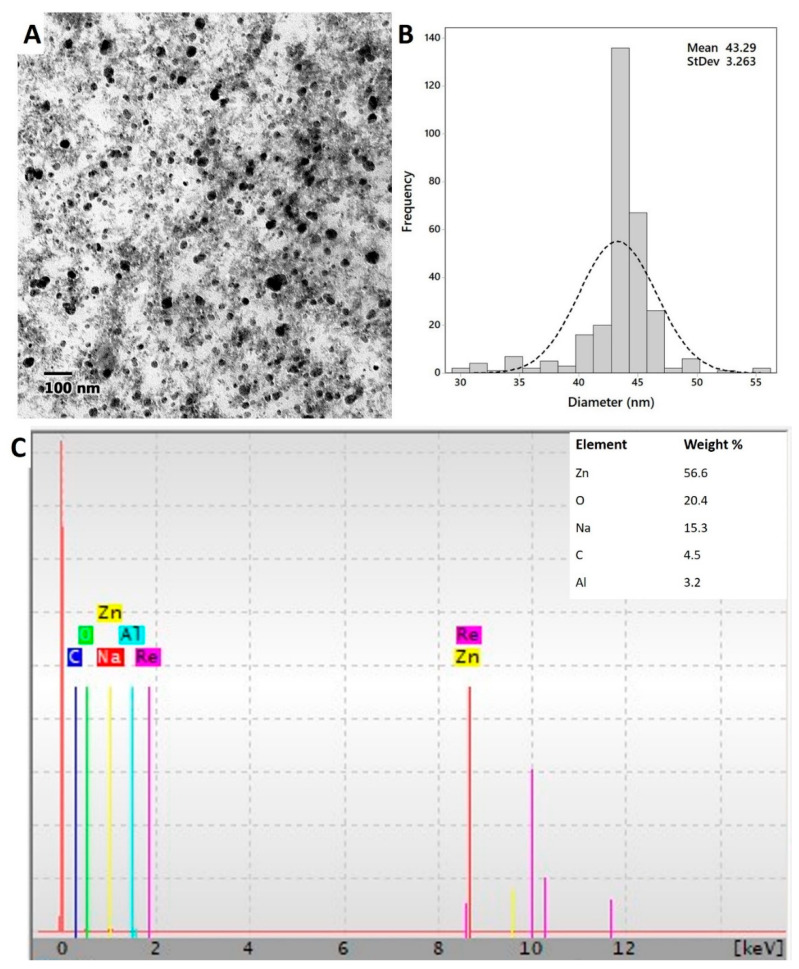
Transmission electron microscope (**A**), particle size distributions (**B**), and energy-dispersive spectroscopy (**C**) for ZnO-NPs synthesized by *A. platensis*.

Figure 4A,B showed the shape, size, as well as size distribution of ZnO-NPs fabricated by *A. platensis*. The TEM image exhibits a good distribution of spherical ZnO-NPs without any aggregation. Moreover, image analysis demonstrated that the size of fabricated ZnO-NPs was in the range of 30.0 to 55.0 nm. Recently, spherical ZnO-NPs were fabricated by *Chlorella* cell extract with the size range of 20.0–50.0 nm [40]. The efficacy of metabolites involved in algal cell extract to fabricate spherical ZnO-NPs was previously investigated [50]. 

The quantitative elemental structure of green synthesized ZnO-NPs was investigated by EDX analysis (Figure 4C). Data analysis revealed that the fabricated ZnO-NPs contain Zn, O, Na, C, and Al with weight percentages 56.6, 20.4, 15.3, 4.5, and 3.2%, respectively. The EDX result confirms the successful fabrication of ZnO through harnessing the metabolites involved in *A. platensis* filtrate; moreover, EDX analysis affirms that Zn and O occupied the major elements in the nanostructure. The presence of other peaks such as C, Na, and Al may be related to the breakdown of capping agents such as polysaccharides, proteins, amino acids, and sugars as a result of X-ray emissions [30]. A recent study confirmed the presence of Zn and O as a major component of ZnO-NPs synthesized by cyanobacterium *Nostoc* sp. EA03 [50]. In addition, Djearamane et al. [51] reported that the main components of ZnO-NPs synthesized by *Spirulina platensis* were Zn and O. 

#### 3.3.4. X-ray Diffraction (XRD) Analysis

**Figure 5:**
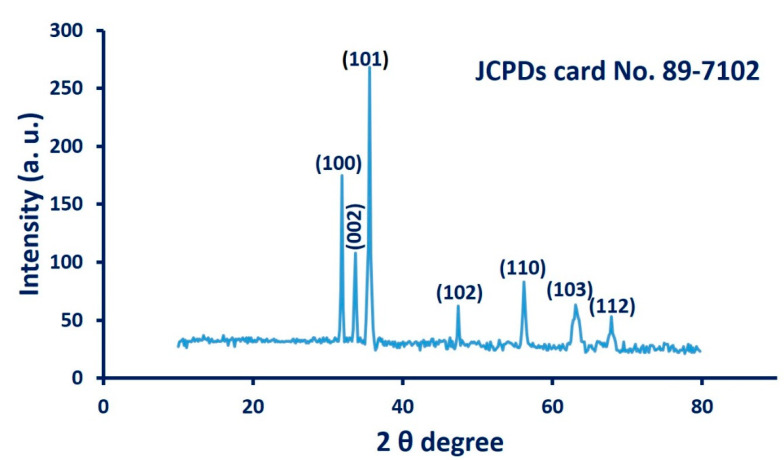
X-ray diffraction (XRD) pattern for ZnO-NPs synthesized by *A. platensis*.

The crystallographic structure of cyanobacterium-mediated biosynthesis of ZnO-NPs was assessed by XRD analysis. As depicted in Figure 5, the formed ZnO-NPs showed seven distinguished peaks at 2 theta degree 31.7°, 34.5°, 36.1°, 47.4°, 56.3°, 63.1°, and 67.9°, which matched to (100), (002), (101), (102), (110), (103), and (112) planes, respectively. All outstanding diffraction peaks in XRD spectra are compatible with those recorded in the Joint Committee on Powder Diffraction Standards (JCPDS, card No. 89-7102), which confirm the crystallographic Wurtzite structure [16,50]. The average crystal size has been calculated from XRD analysis using the Debye–Scherrer equation, which in this study was approximately equal to ≈45 nm. The data from XRD are compatible with those obtained by TEM analysis. The presence of slight peaks in XRD spectra may be related to the crystallization of organic substances that coated the surface of ZnO-NPs [8]. 

#### 3.3.5. X-ray Photoelectron Spectroscopy (XPS) Analysis

**Figure 6:**
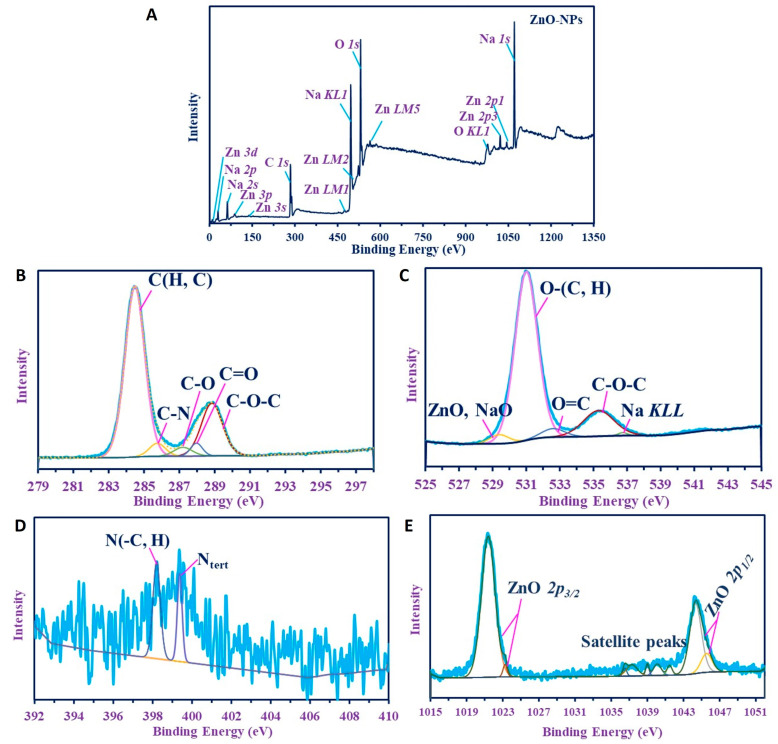
X-ray photoelectron spectroscopy analysis of ZnO-NPs synthesized by *A. platensis. (***A**) Overview survey; (**B**–**E**) denotes C *1s*, O *1s*, N*1s*, and Zn *2p*.

Figure 6A shows the XPS survey spectra of the product. It confirms the composition of the substance by the presence of C, O, and N as C *1s*, O (*1s*, *KL1*), and N *1s*. In addition, the survey analysis revealed the presence of Na as Na (*2s, 2p*, *KL1*), while Zn was confirmed by Zn (*3p*, *3d*, *3s*, *LM1, LM2, LM5*, *2p1,* and *2p3*). 

C *1s* (Figure 6B) was split into five peaks at 284.48, 285.75, 287.9, 287.05, and 288.9 eV for C(H, C), C–N, C–O, C=O, and C–O–C, respectively [52,53], verifying the hydrocarbon composition produced in the medium. N *1s* was deconvoluting into two peaks with low intensities at 398.2 and 399.37 eV for N (C, H) and N_tert_, respectively [53,54]. 

O *1s* (Figure 6C) has more peaks, verifying the structure of the materials; ZnO was overlapped with NaO at 529.4 eV, which confirms the presence of Zn as oxides. In addition, the excess amount of Na in the materials that appeared on the overall spectra is reflected in the O *1s* that have other peaks for Na *KL1* at 536.75 (Figure 6D) [55], while the hydrocarbons peaks have appeared at 531, 532.2, and 535.3 eV for O(C, H), O=C, and C–O–C, respectively [33,52,55]. These results were elucidated also by FT-IR with the broad bands of NH overlapped with OH, C=O, NH, and C–O (see the FT-IR analysis). Zn *2p* (Figure 6E) have several peaks: two for ZnO *2P_3/2_* at 1021.4 eV and 1023.25 eV, another two peaks for ZnO *2p_1/2_* at 1044.2 eV and 1045.55 eV, while satellite peaks verifying the oxide species appeared at 1036.25, 1037.3, 1039, 1040.05, and 1041.65 eV. These spectra indicate the presence of Zn as Zn (II) oxide [56].

### 3.4. Biological Activities of ZnO-NPs Synthesized by A. platensis

#### 3.4.1. Antimicrobial Activity 

**Figure 7:**
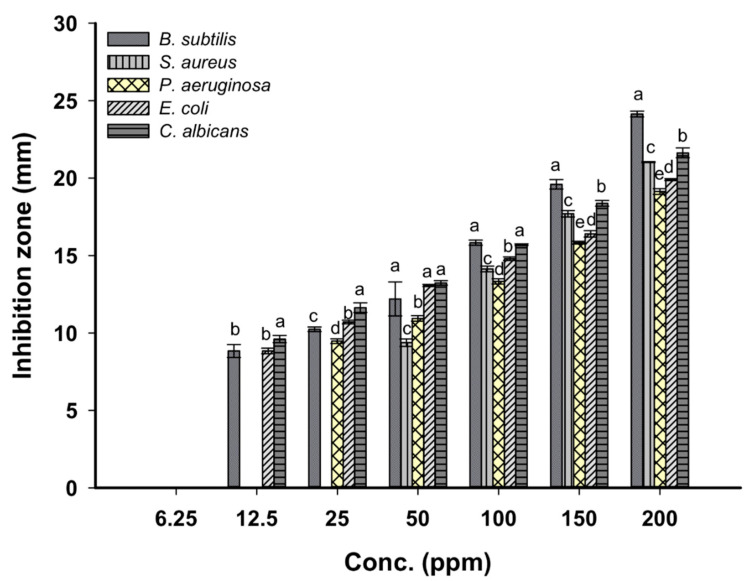
Antimicrobial activities of ZnO-NPs synthesized by *A. platensis* against *Bacillus subtilis, Staphylococcus aureus, Pseudomonas aeruginosa, Escherichia coli,* and *Candida albicans*. Data are statistically different at *p* ≤ 0.05 by Tukey’s test, (*n* = 3); error bars are means ± SE. The different letters, a–e, are signified the significance. Bars with the same letter for each concentration did not differ significantly.

Figure 7 shows the antimicrobial activity of ZnO-NPs synthesized by cyanobacterium *A. platensis* against pathogenic Gram-positive and Gram-negative bacteria as well as unicellular fungi. The obtained data indicated that the antimicrobial activities of NPs are increased with respect to concentration. This phenomenon was completely consistent with previous studies where the activity of Ag, ZnO nanoparticles was dose- and shape-dependent [38,57]. The cyanobacterium cell extract (as control) used to fabricate ZnO-NPs was tested as an antimicrobial agent against bacterial and unicellular fungal used and did not exhibit any activities. At stock colloidal solution (200 ppm), ZnO-NPs exhibit varied activities against *Bacillus subtilis*, *Staphylococcus aureus*, *Pseudomonas aeruginosa, Escherichia coli,* and *Candida albicans* with clear zones of 24.1 ± 0.3, 21.1 ± 0.06, 19.1 ± 0.3, 19.9 ± 0.1, and 21.6 ± 0.6 mm, respectively. As the NPs concentration decreased (50 ppm), the inhibition zone was decreased as well to 12.2 ± 1.9, 9.4 ± 0.4, 10.9 ± 0.3, 13.1 ± 0.1, and 13.2 ± 0.3 mm for *B. subtilis*, *S. aureus*, *P. aeruginosa*, *E. coli,* and *C. albicans,* respectively. The published literature clarifies the antibacterial activity of green synthesized ZnO-NPs against a wide range of pathogenic bacteria such as *Streptococcus pyogenes, S. aureus, P. aeruginosa, E. coli, B. subtilis, Klebsiella aerogenes, Mycobacterium tuberculosis,* and *Proteus mirabilis* [58]. Consistent with our study, spherical ZnO-NPs synthesized by the aqueous extract of *Tabernaemontana divaricate* with a size range of 20 to 50 nm have antibacterial activities against *S. aureus, E. coli,* and *Salmonella paratyphi* [59]. Recently, our study showed that the stock colloidal solution (2000 ppm) of spherical ZnO-NPs synthesized by *Aspergillus terreus* strain AF-1 with a size range of 10 to 45 nm have antibacterial activity against *B. subtilis, S. aureus, E. coli,* and *P. aeruginosa* with diameter inhibition zone ranging between 14.1 ± 0.2 and 20.2 ± 0.2 nm [16]. 

The minimum inhibitory concentration (MIC) is defined as the lowest NPs concentration that inhibits microbial growth [60], and it should be detected for each organism. In this study, the MIC values were 50 ppm for *S. aureus* (9.4 ± 0.4 mm), 25 ppm for *P. aeruginosa* (9.5 ± 0.3 mm), and 12.5 ppm for *B. subtilis, E. coli,* and *C. albicans* recording inhibition zones 8.8 ± 0.7, 8.8 ± 0.3, and 9.6 ± 0.4 mm, respectively. The ZnO-NPs size and their concentrations have critical roles in antimicrobial activities. Several studies have also confirmed that the antibacterial activity of ZnO-NPs is size and concentration-dependent [61]. The smaller NPs size means a large surface area; they penetrate the microbial cell easily through the cell membrane and then enhance antimicrobial efficacy with their high concentration [62]. This phenomenon encourages our finding that correlates between antimicrobial activity and smaller ZnO-NPs size, which in this study was 30.0 to 55.0 nm. 

The toxicity of ZnO-NPs can be attributed to the generation of reactive oxygen species (ROS), the release of Zn (Zn^2+^) ions inside the microbes, and the change in cell wall permeability [63]. The generation of ROS is considered the major reason for nanotoxicity which involved the damage of cellular components (proteins, lipid, nucleic acid, phospholipid, amino acids) [61]. On the other hand, the release of zinc ions (Zn^2+^) has a negative impact on the active transport system, enzymatic reactions, amino acid metabolism, binding to macromolecules, and hence all fundamental microbial functions are inhibited from continuing [60]. The zinc oxides are amphoteric and can react with alkali and/or acids and release Zn^2+^ as shown in the following Equations (4)–(6) [60].

(4) $$\mathrm{ZnO}+2\mathrm{HCl}\;\overset{\mathrm{Acidic}\;\mathrm{medium}\;}{\to }{\mathrm{ZnCl}}_{2}+{\;H}_{2}O$$

(5) $$\mathrm{ZnO}+2\mathrm{NaOH}\;\overset{\mathrm{Alkaline}\;\mathrm{medium}}{\to }{\;\mathrm{Na}}_{2}{\mathrm{ZnO}}_{2}+{\;H}_{2}O$$

(6) $${\mathrm{ZnCl}}_{2}\;\overset{\mathrm{Aquous}\;\mathrm{medium}}{\to }{\mathrm{Zn}}^{2+}+2{\mathrm{Cl}}^{-}$$

The accumulation of ZnO-NPs in the microbial cell membrane is considered another mechanism for nanotoxicity, which correlated with the destruction of the proton motive force and hence changes the plasma membrane permeability. This change causes the rapid discharge of cellular components out of the microbial cell, and then cell viability was reduced [7,61]. 

#### 3.4.2. In Vitro Cytotoxicity Assay 

**Figure 8:**
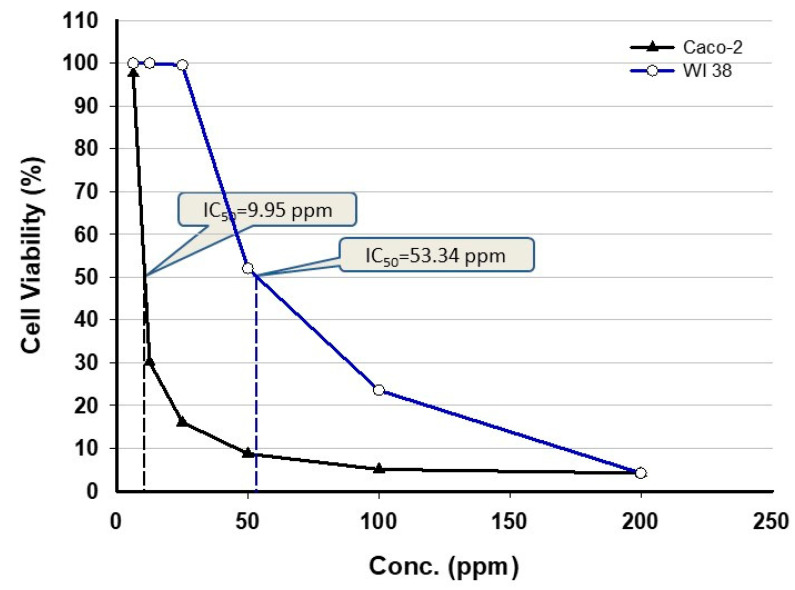
In vitro cytotoxic efficacy of ZnO-NPs synthesized by *A. platensis* against normal (WI 38) and cancerous (Caco-2) cell lines.

Cancer is a life-threatening disease, and finding therapeutic drugs for the treatment of various types of cancer is a challenge. In this study, the cytotoxic efficacy of cyanobacterium-mediated green synthesized ZnO-NPs was evaluated against two cell lines, WI 38 and Caco-2 by using the MTT assay method (Figure 8). The MTT method is a highly accurate and sensitive colorimetric method to investigate the cell viability after exposure to external substances [64]. MTT assay is dependent on the ability of succinate dehydrogenase mitochondrial enzyme to change the tetrazolium yellow dye to formazan crystals, which is directly proportional to cell viability and assayed as optical density [65]. The efficacy of ZnO-NPs in the treatment of cancer cells is interesting, because it is more effective toward proliferative cells as compared to non-proliferative ones [66]. The toxicological efficacy of ZnO-NPs has been investigated against different cell lines [67,68]. Some investigators reported that the ZnO-NPs are toxic to cancerous cell lines only and do not have any cytotoxic effect against normal cells [69,70]. However, the recently little cytotoxic effect of ZnO-NPs was shown toward normal cells as compared to cancerous cells. The ZnO-NPs fabricated by the methanolic extract of *Sargassum muticum* algal species showed toxic effect against cancerous cell lines MCF-7 and MDA-MB-231 and do not exhibit any activity against normal Vero cell; this could be attributed to an activated signal pathway through ligand/receptor interaction [71]. 

Data analysis revealed that the viability of treated cell lines was dose-dependent; as the NPs concentration increased, the viability was decreased. This finding is constant with Rajakumar et al. [72], who reported that apoptosis is correlated with high ZnO-NPs concentration. In this study, the cell viability of WI 83 (normal cell line) and Caco-2 (cancerous cell line) was measured after 48 h and exhibited that the IC_50_ were 53.34 and 9.95 ppm, respectively. According to these results, we can have concluded that the low ZnO-NPs concentration was more effective on cancerous cells as compared to the normal cell line. Ngoepe et al. [73] and Agarwal et al. [74] showed that the cell viability due to ZnO-NPs treatment was assessed after 48 h. With our findings, previous studies proved the efficacy of NPs on cancerous cell viability such as MCF-7 as breast cancerous cell [75], MGC803 as gastric cancerous cell [75], Caco-2 as adenocarcinoma cell [76], and MG-63 as osteosarcoma cell [77]. Data recorded by Malaikozhundan et al. [78] were incompatible with our study, which showed that spherical ZnO-NPs with size 30–40 nm synthesized by seed extracts of *Pongamia pinnata* can reduce the cell viability of cancerous cell MCF-7 at a concentration higher than 50 ppm. On the other hand, ZnO-NPs (with size range 20–50 nm) synthesized by the aqueous extract of algal species *Gracilaria edulis* have dose-dependent cytotoxic efficacy against SiHa (Human cervical cancer) cell line with an IC_50_ value of 35 ± 0.03 ppm [67]. 

Interestingly, ZnO-NPs are utilized widely in cancer therapy and can reduce the proliferation of cancer cells. Some of the published literature reported that ZnO-NPs are nontoxic and biocompatible [79], while others have reported both the in vivo and in vitro toxicity of the ZnO-NPs, particularly on mammalian cells [80]. According to data obtained in this study and others recently published, it can be clarified that the toxicity of ZnO-NPs is concentration-dependent. On the other hand, the treatment of leukemic T cells, cancerous cells, and inhibited pathogenic growth can be correlated with the toxicity of ZnO-NPs [81,82]. Other advantages correlated with ZnO-NPs include overcoming the drug-resistant problems within the pharmaceutical industry because of the non-selectivity of ZnO-NPs [83]. The biological activities of ZnO-NPs are correlated with their physicochemical properties as well as the dispersion characteristics of NPs. In some cases, the NPs agglomerate or aggregate when reacting with physiological fluids; therefore, the study of the dispersion efficacy of NPs is a critical factor to detect in vitro and in vivo cytotoxicity [84]. The addition of dispersant is an important factor to modify the physical and thermal properties of the NPs such as conductivity and viscosity [85]. The addition of dispersants was helpful in heat resistance in heat transfer applications and helped in foam formation during the heat and coal process [86].

## 4. Conclusions

The green synthesis of ZnO-NPs using microalga *Arthrospira platensis* has received prodigious interest because of their rapid growth, high biomass production, environmentally safe nature, and low-cost protocol. In this study, the microalgae *A. platensis* was isolated from the water sample and subjected to microscopic as well as molecular identification. The biosynthesized ZnO-NPs were characterized by UV-Visible spectroscopy, FT-IR, TEM, EDX, XRD, and XPS analyses. The obtained data showed the successful fabrication of spherical ZnO-NPs with a size range of 30.0 to 55.0 nm at a maximum wavelength of 370 nm. The functional groups present in biomass filtrate have critical roles in the fabrication process as shown in FT-IR. Moreover, the crystallographic structure was confirmed by XRD. The XPS spectra indicated the presence of Zn as Zn (II) oxide. The biological activities including antimicrobial and in vitro cytotoxicity were also the main goals. Data showed that the activities of biosynthesized ZnO-NPs were dose- and time-dependent. The biosynthesized ZnO-NPs exhibit varied activities against *B. subtilis*, *S. aureus*, *P. aeruginosa*, *E. coli,* and *C. albicans* with clear zones ranging between 19.1 ± 0.3 and 24.1 ± 0.3 mm. Moreover, the in vitro cytotoxic effect of ZnO-NPs against normal (WI 38) and cancer (Caco-2) cell lines was investigated. Data exhibit that the IC_50_ values were 53.34 and 9.95 ppm for normal and cancer cell lines, respectively. The obtained data confirm the high efficacy of cyanobacterium *A. platensis* as a biocatalyst for the green synthesis of ZnO-NPs for integration into different biomedical applications.

## Data Availability

The data presented in this study are available on request from the corresponding author.
